# TaqMan qPCR for Quantification of *Clonostachys rosea* Used as a Biological Control Agent Against *Fusarium graminearum*

**DOI:** 10.3389/fmicb.2019.01627

**Published:** 2019-07-16

**Authors:** Alejandro Gimeno, Elina Sohlberg, Tiina Pakula, Jenni Limnell, Beat Keller, Arja Laitila, Susanne Vogelgsang

**Affiliations:** ^1^Ecological Plant Protection in Arable Crops, Research Division Plant Protection, Agroscope, Zurich, Switzerland; ^2^Molecular Plant Biology and Phytopathology, Department of Plant and Microbial Biology, University of Zurich, Zurich, Switzerland; ^3^VTT Technical Research Centre of Finland Ltd., Espoo, Finland

**Keywords:** qPCR, *Clonostachys rosea*, biological control agent, *Fusarium graminearum*, Fusarium head blight, MycoKey

## Abstract

*Clonostachys rosea* is a biological control agent against *Fusarium graminearum* in small grain cereals and maize. Infections with *F. graminearum* do not only reduce the yield but, due to the production of mycotoxins, also affect the entire value chain of food and feed. In addition, production of other secondary metabolites such as hydrophobins, also known as gushing inducers, may cause quality challenges for the malting and brewing industry. Sustainable disease control strategies using *C. rosea* are treatment of infected residues of the previous crop, direct treatment of the actual cereal crop or post-harvest treatment during malting processes. Follow-up of growth and survival of biocontrol organisms during these different stages is of crucial importance. In the current study, we developed a quantitative real-time PCR detection method that amends the currently available culture-dependent techniques by using TaqMan chemistry with a highly specific primer and probe set, targeting the actin gene. We established a sensitive assay that detects the biological control agent down to 100 genome copies per reaction, with PCR efficiencies between 90 and 100%. The specificity of the assay was confirmed against a panel of 30 fungal and 3 bacterial species including 12 members of the Fusarium head blight complex and DNA of barley, maize and wheat. The DNA of *C. rosea* was detected in *Fusarium*-infected maize crop residues that were either treated in the laboratory or in the field with *C. rosea* and followed its DNA throughout the barley malting process to estimate its growth during grain germination. We used a standardized DNA extraction protocol and showed that *C. rosea* can be quantified in different sample matrices. This method will enable the monitoring of *C. rosea* during experiments studying the biological control of *F. graminearum* on cereal crop residues and on cereal grains and will thus contribute to the development of a new disease control strategy.

## Introduction

*Clonostachys rosea* is a mycoparasitic fungus able to attack many important plant pathogens in the rhizosphere and the phyllosphere including different *Fusarium* species (Jensen et al., 2000; Xue, 2003, Xue et al., 2009). Currently, there are two described infraspecific forms, *C. rosea* f. *rosea* (formerly *Gliocladium roseum*) and *C. rosea* f. *catenulata* (formerly *Gliocladium catenulatum*) that are found in soils living as parasites and decomposers (Schroers, 2001). Both forms are extensively described as potential biological control agents (BCA). In fact, *C. rosea* is marketed as a natural fungicide or biostimulant for its ability to antagonize pathogens, induce plant resistance and promote vigor under different trade names using viable conidia of the fungus as the active ingredient (e.g., Prestop^®^ with *C. rosea* f. *catenulata* strain J1446 or Endofine^®^ with *C. rosea* f. *rosea* strain 88–710).

*Clonostachys rosea* is well recognized for its ability to antagonize the mycotoxin producing fungus *Fusarium graminearum* (Schöneberg et al., 2015), the predominating causal agent of Fusarium head blight (FHB) of wheat and barley (Goswami and Kistler, 2004; Dweba et al., 2017). *F. graminearum* is one of the most important plant pathogens worldwide (Dean et al., 2012) and its ability to produce the toxic type B trichothecene deoxynivalenol (DON) and the mycoestrogen zearalenone (ZEN) (Desjardins, 2006) adds a dimension to the FHB disease problem that extends into the sectors of public health and the value chain of food and feed production. Considering the negative impacts on growers and buyers, Wilson et al. (2018) estimated the economic loss caused by FHB and DON contamination including the cost for risk mitigation solely in the United States between 2016 and 2017 at US$1.47 billion for wheat and barley.

In the field, the most important driver for a FHB epidemic is the presence of fungal inoculum from residues of previous crops. In the soil and on the surface, *F. graminearum* survives saprotrophically on the residues as mycelium, sporodochia, or chlamydospores and develops perithecia that produce wind-dispersed ascospores, infecting the host crop together with rain-dispersed conidia (Leplat et al., 2013). Against this background, considerable success has been reported exploiting the antagonist *C. rosea* to reduce the survival of *F. graminearum* on infected crop residues, especially in wheat and maize (Luongo et al., 2005; Palazzini et al., 2013; Schöneberg et al., 2015). In addition, significant reduction of DON contamination in grain by up to 33% were reported after application of *C. rosea* strain ACM941 onto the heads of flowering wheat (Xue et al., 2014). Thus, biological control of *F. graminearum* would be a valuable addition to the available pre-harvest measures like crop rotation, tillage, cultivar resistance, forecasting systems or chemical control that are often not sufficient to control FHB (Wegulo et al., 2015). Another much less understood opportunity is the application of *C. rosea* on *Fusarium*-contaminated grain in the post-harvest process of barley malting for beverage production. In previous studies, it was shown that the use of antagonistic yeasts and lactic acid bacteria during malting could restrict the growth of indigenous *Fusarium* fungi during the process, which reduces the negative impacts on the final malt quality such as mycotoxin contamination and accumulation of beer gushing inducers (Laitila et al., 2002, 2007). In the case of *C. rosea*, the ability to degrade ZEN by enzymatic activity as part of the antagonistic interaction with *F. graminearum* further suggests the possibility to use it for bioremediation of infected grain lots during industrial processes (Kosawang et al., 2014).

Relatively little is known about the survival and distribution of *C. rosea* after the application and available quantification methods are based on laboratory cultivation techniques (Pan et al., 2013) or on strain-specific molecular markers currently used for quantitative real-time polymerase chain reaction (qPCR) detection in soils (Legrand et al., 2018). Hence, for the monitoring of crop residues and grains, a widely applicable DNA based quantification method on the species level is needed. *C. rosea* may compete against the pathogen in different plant tissues, making it difficult and time-consuming to detect and quantify this antagonist. Currently, DNA extract preparation is relatively cheap, fast and reproducible from a wide range of environmental samples. Together with the accurate and sensitive nature of qPCR, this is a preferential approach for high throughput detection of pathogens and antagonists. It can enhance the understanding of the growth dynamics of fungi that are influenced by environmental conditions, the application strategy and agricultural or industrial practices (Sanzani et al., 2014).

The main objective of the current study was to develop new primers and a qPCR assay for the detection, quantification and monitoring of both forms of *C. rosea* used as a BCA against *F. graminearum* on crop residues and in malting barley. We selected the commonly conserved region encoding the actin gene for identification of sequences unique to *C. rosea* and further enhanced the accuracy by using TaqMan chemistry. Finally, we evaluated the newly established assay for efficient and specific quantification of target DNA in extracts from pure cultures and environmental samples. This evaluation included a wide range of non-target species and amplification in samples from crop residues and grain taken from the laboratory, the field and from a small-scale malting trial where *C. rosea* was applied against *F. graminearum*.

## Materials and Methods

### Microbial Isolates

The microbial isolates used in this study were obtained from the culture collection of the VTT Technical Research Centre of Finland (VTT Culture Collection, Espoo, Finland), the Westerdijk Fungal Biodiversity Institute (CBS, Utrecht, Netherlands) and the Culture Collection of Switzerland (CCoS, Wädenswil, Switzerland) (Table 1).

**TABLE 1:** Fungal and bacterial isolates and their origins.

**Species name**	**Strain ID**	**Origin/host, Country**
*Clonostachys rosea* f. *rosea*	CCOS 1865	Agricultural soil, Switzerland
	CCOS 1864	Agricultural soil, Switzerland
	VTT D-161647	Field pea, Canada
	Strain 016 (J. Köhl,	Unknown, Netherlands
	Wageningen University)	
	VTT D-97674	Agricultural soil, Finland
	VTT D-96593	Paper mill, Finland
*Clonostachys rosea* f. *catenulata*	VTT D-97673	Agricultural soil, Finland
	VTT D-95548	Recycled fiber pulp, Finland
*Clonostachys byssicola*	CBS 364.78	Bark, Venezuela
*Clonostachys pseudochloroleuca*	CBS 187.94 T	Palm frond, French Guiana
*Clonostachys rhizophaga*	CCOS 1863	Soil, Switzerland
	CBS 125416	Bamboo, Italy
*Clonostachys rogersoniana*	CBS 920.97 T	Soil, Brazil
*Fusarium avenaceum*	VTT D-80141	Barley, Finland
*Fusarium cerealis*	VTT D-96601	Barley, Finland
*Fusarium culmorum*	11132 (Agroscope)	Wheat, Switzerland
*Fusarium equiseti*	VTT D-82087	Muskmelon, Turkey
*Fusarium graminearum*	CBS 121292	Wheat, Switzerland
	2113 (Agroscope)	Wheat, Switzerland
	1145 (Agroscope)	Wheat, Switzerland
	VTT D-95470	Maize, Europe
	VTT D-82082	Barley, Finland
	VTT D-80148	Barley, Finland
	VTT D-76038	Barley, Finland
	VTT D-82182	Oat, Finland
*Fusarium langsethiae*	VTT D-03931	Barley, Finland
*Fusarium oxysporum*	VTT D-80134	Wheat, Europe
*Fusarium poae*	335 (Agroscope)	Wheat, Switzerland
*Fusarium sambucinum*	VTT D-77056	Cereal grain, Europe
*Fusarium solani*	VTT D-77057	Cereal grain, Europe
*Fusarium sporotrichioides*	VTT D-72014	Maize, Europe
*Fusarium tricinctum*	VTT D-131559	Barley, Finland
*Microdochium majus*	VTT D-94433	Wheat, Switzerland
*Microdochium nivale*	VTT D-131555	Barley, Finland
*Acremonium polychronum*	VTT D-96653	Moldy house, Finland
*Alternaria alternata*	VTT D-76024	Barley, Finland
*Aspergillus clavatus*	VTT D-94422	Malted barley, Finland
*Aspergillus ochraceus*	VTT D-00808	Barley, Finland
*Aureobasidium pullulans*	VTT D-071272	Wheat, Finland
*Cochliobolus sativus*	VTT D-76039	Barley, Finland
*Curvularia inaequalis*	VTT D-79121	Barley, Turkey
*Epicoccum nigrum*	VTT D-76046	Unknown, France
*Eurotium amstelodami*	VTT D-03923	Barley, Finland
*Geotrichum candidum*	VTT D-94425	Malted barley, Finland
*Penicillium vermoesenii*	VTT D-051089T	Lemon, Spain
*Penicillium verrucosum*	VTT D-99750	Barley, Denmark
*Rhodotorula glutinis*	VTT C-92011	Malted barley, Finland
*Trichoderma harzianum*	VTT D-161648	Soil, Italy
*Trichothecium roseum*	VTT D-76042	Barley, United Kingdom
*Pantoea agglomerans*	VTT E-90398	Barley, Finland
*Lactobacillus plantarum*	VTT E-78076	Malting process, United Kingdom
*Leuconostoc citreum*	VTT E-93497	Malting process, Finland

With respect to species specificity, 46 different fungal isolates, including two yeast fungi, were included in the evaluation of the method. Isolates were cultured on potato dextrose agar (all fungi except of yeasts: CM0139, Oxoid Ltd., Hampshire, United Kingdom) or yeast mold agar (yeast: B271210, Becton, Dickinson and Company, NJ, United States) and incubated for 2–4 days at 25°C in a 12/12 h ambient light (36W/21-840 Lumilux Plus; Osram Gmbh, Munich, Germany)/dark rhythm before the DNA was isolated. Furthermore, eight isolates of *C. rosea* were applied, including the strains 016 and ACM941 (VTT D-161647), previously described as BCAs of *F. graminearum* (Xue et al., 2009; Schöneberg et al., 2015). Other species within the genus *Clonostachys* were *C. rhizophaga* (Tehon and Jacobs), *C. byssicola* (Schroers, stat. nov.) *C. pseudochloroleuca* (Schroers, stat. nov.) and *C. rogersoniana* (Schroers, stat. nov.). The diverse fungal panel also included members of the FHB-disease complex (19 *Fusarium* spp. strains and two *Microdochium* spp. strains), other fungal antagonists (*Trichoderma harzianum* and *Aureobasidium pullulans*), common saprophytes and other pathogens, all related to the agricultural production, biological control or post-harvest processing of wheat, barley and maize.

Bacterial isolates were used exclusively in the evaluation of the primer specificity. The included species were endophytic *Pantoea agglomerans* from barley grain as well as the BCA *Lactobacillus plantarum* and *Leuconostoc citreum*, originally isolated from the beer malting process by Laitila et al. (2002). *Pantoea agglomerans* was cultivated on PCA agar (Plate count agar, Difco Inc., Detroit, United States) and lactic acid bacteria were cultured on MRS agar (de Man, Rogosa and Sharpe, CM0361, Oxoid Ltd., Hampshire, United Kingdom) at 25°C in a 12/12 h ambient light/dark rhythm for 2 days before DNA isolation.

### DNA Isolation

DNA from plant and microbial samples was isolated using the FastDNA Spin Kit for Soil (MP Biomedicals LLC, Solon, United States) according to the manufacturer’s instructions. For the cell lysis, a FastPrep-24 benchtop homogenizer (MP Biomedicals) was used for 2 × 60 s at 6 m/s with a 5 min cooling period on ice between runs. To obtain fresh cell material for isolation from pure cultures, both fungi and bacteria were grown on their respective growth media with a sterilized layer of cellophane or filter paper placed between agar surface and microbe. The cell material was then carefully scraped off using one-way sterile stainless steel blades (Feather Safety Razor Co., Ltd., Osaka, Japan). The material was either immediately used for isolation or first frozen in liquid nitrogen and subsequently lyophilized using a benchtop vacuum centrifuge (CentriVap; Labconco Corporation, Kansas City, MO, United States). The isolated DNA was eluted in 100 μl of DNase and pyrogen-free water before being stored at −20°C in aliquots of 50 μl or at 4°C between experiments. The DNA concentration and the absorbance ratio at 260/280 nm were determined with Nanodrop (2000/2000c; Thermo Fisher Scientific, Waltham, MA, United States).

### Design of Primers and the Hydrolysis Probe

Primers *VTTact*-f and *VTTact*-r were designed *in silico*, by searching for target specific and single copy regions in close vicinity of the conserved coding sequences of the actin gene (Table 2). The sequence of *C. rosea* CBS 125111 actin gene (gene ID 114937) was obtained from the JGI database (Joint Genome Institute^1^). In order to identify non-conserved areas, the gene sequence (including introns, 5′ and 3′ untranslated regions and additional 200 bp extension to both 5′ and 3′ direction) was first used as a query in a BLASTn search against the NCBI nucleotide collection (nr/nt^2^). The sequences of the ten closest homologs revealed by the BLASTtn search were aligned using ClustalO^3^. Several unique primers binding to the non-conserved regions were designed using Primer-BLAST and the NCBI nr database^4^. The primers were designed to amplify a 150–200 bp region in the gene and the expected melting temperature of the primers was 60–63°C. The final selection of *VTTact*-f and *VTTact*-r was based on the alignment of the predicted amplicon sequence from JGI_CBS 125111 (scaffold_2: 1642573–1642766) with the available genome sequences for *C. rosea* strains IK 726 (Karlsson et al., 2015) and YKD 0085 (Liu et al., 2016) to reveal possible differences between isolates of the same species. Using the predicted amplicon sequence (156 bp), the design of the hydrolysis probe for the TaqMan PCR (Table 2) was conducted with the sequence analysis software Geneious^5^. The primers and the probe were synthesized by Integrated DNA Technologies (IDT Inc., Coralville, United States). To test the specificity with environmental samples, microbial DNA extracts as well as DNA isolated from sterile barley tissue culture were freshly prepared and diluted to a template concentration of 2 ng μl^–1^. For each extract, two individual reactions were performed using 10 ng of total DNA (5 μl) and the amplification was examined. PCR products were further loaded on 1.5% (w/v) agarose gels stained with Midori Green Nucleic Acid Stain (Nippon Genetics Europe, Düren, Germany) and using a DNA ladder (GeneRuler, Thermo Fisher Scientific) to estimate the product size by electrophoresis. Products of the *C. rosea* strains 016 and SHA77.3 were sent to Microsynth AG (Balgach, Switzerland) for purification and both-end Sanger sequencing.

**TABLE 2:** Primer and hydrolysis probe sequences (5′–3′) for the specific amplification of *Clonostachys rosea* with TaqMan qPCR.

**Primer/probe**	**Sequence (5′ to 3′)**
*VTTact*-forward	GGCCAGAGATTGTGTTGATGA
*VTTact*-reverse	ACAGGTTAGGCTCAATGCTC
*VTTact* probe	GAGGCTGGCAAGAGAGGTCAGTCAC

### TaqMan qPCR

All qPCR reactions were performed on a Lightcycler 480 II real-time PCR instrument (Roche Diagnostics Ltd., Risch-Rotkreuz, Switzerland) using the corresponding software (release 1.5.0. Version 1.5.0.39) in white Multiwell 96-well plates and sealed with adhesive foil (04729692001; 04729757001, Roche Molecular Systems Inc., Pleasanton, United States). The combination of the primers with the hydrolysis probe was evaluated using the Lightcycler 480 Probes Master TaqMan chemistry (Roche Molecular Systems Inc.) in 20 μl volume reactions, containing 2× Probes Master, 6 pmol of each primer, 2 pmol of the hydrolysis probe and 5 μl of template. The PCR program consisted of a pre-incubation for 10 min at 95°C, followed by 40 cycles of denaturation for 10 s at 95°C, annealing for 30 s at 62°C and extension for 1 s at 72°C including signal detection. The run was finalized with a cooling period of 10 s at 40°C.

### Standard Curve

For the preparation of the standard curve, a sequence verified synthetic DNA fragment (gBlocks gene fragment) comprising the amplified region and additional 50 bp to both 5′ and 3′ direction according to the sequence of the *C. rosea* isolate JGI_CBS 125111 was manufactured by IDT Inc. (Table 3). The linear dsDNA fragment was suspended in molecular grade water (W4502-1L, Sigma-Aldrich, St. Louis, United States) to a concentration of 10 ng DNA μl^–1^ and stored at −20°C between experiments. The initial stock solution contained 3 × 10^10^ target copies μl^–1^, which was calculated by converting the stock concentration (ng DNA μl^–1^) and the mass of the fragment (determined and provided by IDT Inc.) into copy numbers according to the manufacturer’s instructions. The standard curve was prepared in tenfold dilutions over a range of 10 to 10^6^ target copies per reaction and each standard was measured in three technical replicates.

**TABLE 3:** Sequence of the synthetic DNA fragment used in the preparation of the standard curve (5′–3′).

**Synthetic DNA fragment (gBlocks gene fragment) sequence (5′ to 3′)**
GTCACCGACGTAGGAGTCCTTCTGGCCCATACCAATCATGATACTGCCAA**ACAGGTTAGGCTCAATGCTC**TCAGTTATGGAAGCTCCCCCGATAAGGGGTCGCTCTGG TCAATTCGGCATTTCCAACTTACCCATGGTGACGGGGACGACCGACAATGGAGGCTGGCAAGAGAGGTCAGTCACAA**TCATCAACACAATCTCTGGCC**AGCATGGCGATTGTGCTGGCAGCGCAAGGGGCATCAAAGTGGGGTACTCA

*The locations of the primer binding sites are marked in bold and underlined.*

### Evaluation of PCR Inhibition

To estimate possible PCR inhibitory effects of co-extracted molecules from cereal samples, the method of Schneider et al. (2009) was adapted to be used with three different cereal DNA matrices. First, DNA was isolated from three types of plant tissues: maize stalks, barley grains and wheat grains harvested from mature plants and dried for 96 h at 30 ± 2°C. The plant DNA was isolated from 100 mg finely ground material (MM400; Retsch GmbH, Haan, Germany) and several dilutions were prepared. The evaluation was done with increasing amounts of plant DNA, resulting in total inputs of 0 (control), 1, 10, 25, 50, and 100 ng plant DNA per PCR reaction. Each individual reaction, spiked with 10^6^ copies of the synthetic DNA fragment, was performed in triplicates under the qPCR conditions described above. PCR inhibition was determined by the comparison of the cycle threshold (Ct) values between the reactions spiked with an equal amount of target copies and mixed with increasing amounts of plant DNA for each of the three plant-derived sample types.

### Experimental Samples

To validate the method and demonstrate possible applications of the presented method, two distinct sets of samples were obtained from experiments on the pre- and post-harvest control of *Fusarium* spp.

#### Maize Crop Residues – Sample Set 1

Maize stalks were taken from a conventionally managed silage maize field located in Switzerland after harvest and were subsequently infected in the laboratory with a conidial suspension of *F. graminearum* isolate 0410 (CBS 121292) to simulate infected crop residues present in the field. The method for inoculation was adapted from Schöneberg et al. (2015) for maize stalks instead of wheat straw. Stalks were cut to a length of 8 cm, split in half lengthwise with one node per piece and sterilized by autoclaving twice for 15 min at 121°C under pressure. The volume of the *F. graminearum* conidia suspension [2 × 10^5^ conidia/ml in sterile water with 0.02% Tween20 (Riedel-de-Häen, Sigma-Aldrich GmbH, Seelze, Germany)] was increased from 40 to 800 ml to allow for complete immersion using a 1000 ml beaker. Following the inoculation, one halve of the infected stalks were distributed between wheat rows on bare soil in April 2018 at an experimental plot of the federal agricultural research station of Agroscope in Zurich, Switzerland. The other half was simultaneously incubated in Petri dishes (∅ 14.5 cm) on saturated and sterilized vermiculite at 18 ± 2°C and 40 ± 2% relative humidity in a 12/12 h NUV (black light blue tubular fluorescent lamps, wavelength 365 nm)/ dark rhythm. Two days after pathogen inoculation, the biological control strains *C. rosea* 016, NBB2.9 (CCOS 1865) and SHA77.3 (CCOS 1864) were applied individually to the infected stalks by complete immersion for 5 min in 400 ml of conidial suspension of the respective *C. rosea* strain (10^7^ conidia/ml with 0.02% Tween20). In both, field and laboratory, the distribution of the stalks/Petri dishes followed a completely randomized block design with four replicates (blocks). To quantify the number of *C. rosea* copies per ng of DNA with qPCR, extracts were prepared from the treated residues 10 weeks post inoculation (Supplementary Figures S1, S2). The maize stalk pieces were lyophilized and then ground to a fine powder under liquid nitrogen with a benchtop mixer mill (MM400; Retsch GmbH, Haan, Germany). Subsequently, the total DNA was extracted from a 50 ± 2 mg subsample of the homogenized powder. The negative control samples were extracts from untreated maize stalks while DNA of maize stalks treated solely with *C. rosea* strain 016 served as positive controls. Undiluted DNA extracts were stored at −20°C until the measurement. The PCR conditions and reagents were as described above. For the measurements, the samples were diluted 1:10 and the four biological replicates were analyzed in two repeated measurements.

#### Barley Grains – Sample Set 2

Barley grain samples inoculated with *C. rosea* were obtained from an experiment where the BCA was applied for the first time to malting barley grains (VTT Technical Research Centre of Finland Ltd.). The aim was to verify whether the BCA, associated with barley and wheat does not have any negative impact on grain germination properties and on the final quality of the malt. Malting trials were carried out with two different barley batches of 1 kg each from the crop season 2016/2017 obtained from BOORTMALT, Minch Malt Ltd., Athy, Kildare, Ireland.

Two batches, TS17-1 and TS17-19, of the barley variety “Irina” (KWS Saat SE, Einbeck, Germany) showing high (3.15 ± 0.18 pg ng^–1^) and low (0.70 ± 0.01 pg ng^–1^) levels of natural contamination with *Fusarium* sp. DNA, respectively, were processed. The grain samples were malted in a computer controlled micromalting equipment (Hulo Engineering, Helsinki, Finland) with a separate drum for each sample, as described by Virkajärvi et al. (2017). Prior to steeping, the grain samples were inoculated with the *C. rosea* strain ACM941 at 10^6^ conidia/kg in a volume of 10 ml sterile water and incubated at 25°C in the dark for 24 h (moisture content 30%) in order to activate the BCA. Subsequently, grains were steeped at 16°C with alternating wet steep and air rest periods (first steep 6 h, air rest 16 h, and second steep 5 h) to a moisture content of 45 ± 1%. The steeped barley was germinated for 5 days at 16°C. Finally, the barley was kilned in warm air (start at 16°C) for 21 h with a stepwise temperature increase up to 85°C in a separate kiln. The moisture content after kilning was approximately 4%. The first sampling took place after the activation of *C. rosea* and subsequently after the completion of each malting stage. The controls were treated with sterile water. Total DNA extracts were prepared in three replicates from 100 ± 2 mg subsamples of ground material prepared from 20 g malted grain with the FastDNA Spin Kit (Supplementary Figure S3). The PCR conditions and reagents were as described above and the three biological replicates were analyzed in two repeated measurements.

### Data Analysis

The Lightcycler 480 Software (Version 1.5.0.39) was used to obtain the Ct values, the efficiencies as well as the calculated numbers of target copies. The pre-programmed Abs Quant/2nd Derivate Max method was used to analyze the results and the number of target copies was normalized over the total amount of genomic DNA in the template. The statistical analysis of the data was performed with SPSS Statistics (Version 24) for Windows 10. Mean values, standard deviations, figures and tables were calculated with Excel 2016 for Windows 10.

The *r*^2^ values of the standard curve amplifications were obtained from linear regressions between Ct values and the log-transformed number of target copies. Significantly inhibiting effects of plant DNA on qPCR were analyzed by comparisons between Ct values of plant DNA containing reactions to the control reactions by performing multiple pairwise *t*-tests (α = 0.01). The analysis of the experimental samples was done separately for the DNA extracts of maize stalk and malting barley. Maize stalk samples were first separated into laboratory and field samples and then each analyzed by one-way-analysis of variance (ANOVA) on the response variable “copies per ng DNA” with “treatment” as the predicting factor (α = 0.05). *Post hoc* multiple comparisons were performed using the Tukey test (α = 0.05). The normal distribution of residuals and the homogeneity of variance were verified, using Shapiro–Wilk’s method and Levene’s test, respectively. For the malting samples, the assumptions of normal distribution of residuals and the homogeneity of variance were not met, hence, they were separated into the different barley batches and the data were analyzed by ANOVA on ranks using the Kruskal–Wallis test. The response variable was “copies per ng DNA” and the predicting factor was “malting stage” with a significance level of 0.05. *Post hoc* separation was done by a stepwise multiple comparison using the method of Campbell and Skillings (1985).

## Results

### Amplification Specificity

The evaluation of 46 fungal, three bacterial and plant DNAs by qPCR confirmed the highly specific amplification of the newly developed TaqMan assay. For all *C. rosea* isolates (including *C. rosea* f. *rosea* and *C. rosea* f. *catenulata*), the amplification produced a single product with a size of 150 bp. Both-end Sanger sequencing of purified PCR products and the sequence alignment further confirmed the identity of the amplicons (GenBank Accession Number: MN052804 and MN052805). The wide range of other fungi related to cereal hosts such as members of the FHB-disease complex (19 *Fusarium* spp. and two *Microdochium* spp.), other fungal antagonists (*T. harzianum* and *A. pullulans*), common saprophytes and other pathogenic species did not amplify. Furthermore, amplification of bacterial or plant DNAs was not detected. The assay differentiated *C. rosea* from the closely related species *C. rogersoniana* by the presence or absence of detection, respectively, but not against two tested *C. rhizophaga* isolates (CCOS 1863 and CBS 125416) and *C. pseudochloroleuca* isolate CBS 187.94T that amplified at the same cycle. The *C. byssicola* isolate CBS 364.78 was certainly differentiated by its later amplification (+10 cycles compared with *C. rosea*), but 10 ng of DNA per reaction were still detected within the considered Ct range. Amounts <1 ng DNA per reaction of isolate CBS 364.78 were no longer detected (Supplementary Figure S4 and Supplementary Table S1).

### Sensitivity and Efficiency

**FIGURE 1:**
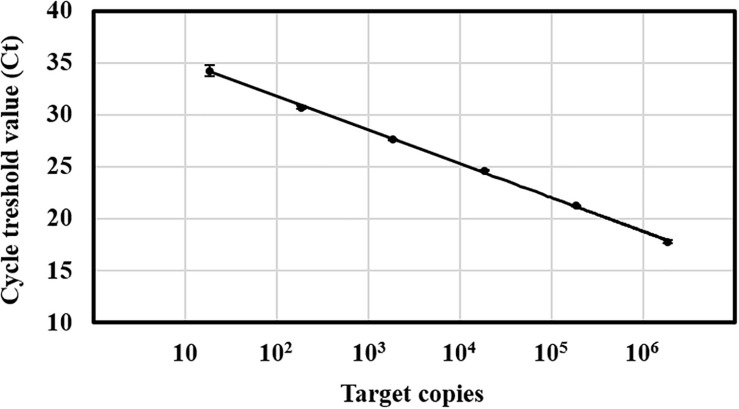
Standard curve of the TaqMan qPCR for *Clonostachys rosea*. Target copies against the cycle threshold (Ct) values of one single qPCR run. Target range was from 18 to 1.8 × 10^6^ copies per reaction. The number of target copies on a log-scaled *X*-axis were plotted against the Ct values from 10 to 40 on the *Y*-axis. Linear regression equation of the standard curve was *Y* = –3.38x + 38.11 at *r^2^* = 0.99. The efficiency was 99% over five orders of magnitude. Vertical bars represent the standard deviation of three technical replicates.

The evaluation of the Ct values from the standard curve amplification revealed a linear dynamic range from 10 to 10^6^ target copies, corresponding to a Ct range of 35∼18 cycles (Figure 1). The lower limit of detection of *C. rosea* was determined around 100 target copies per reaction as 35 cycles was set to be the cutoff value for the method due to uncertainty of the last five cycles. In comparison, the detection of genomic DNA isolated from *C. rosea* strain SHA77.3 was possible down to 1 pg DNA per reaction (Supplementary Figure S5). Linear regressions between the log-transformed number of target copies and the corresponding Ct values revealed *r*^2^ values > 0.99 while the mean efficiency of the PCR was 94 ± 4% standard deviation (STD) (*n* = 15 qPCR runs). No PCR inhibition was observed when different amounts of plant DNA isolated from wheat and barley grains or maize stalks were added to the qPCR in increasing concentrations from 1, 10, 25, 50, to 100 ng. The multiple pairwise *t*-tests revealed no significantly different Ct values compared with the control. Overall, the mean Ct values ± STD were 18.7 ± 0.09, 18.7 ± 0.04, and 18.7 ± 0.05 cycles for maize stalk, barley grain, and wheat grain DNA, respectively.

### Detection in Experimental Samples

Overall, 96 different DNA samples (from 48 maize stalk and 48 malted barley grain batches) were analyzed in two repeated measurements. Among all, 60% of the samples showed a positive signal above the limit of detection within an amplification range between 22 and 35 cycles. In both sample sets*, C. rosea* was always detected when applied and not detected in the negative control samples.

**FIGURE 2:**
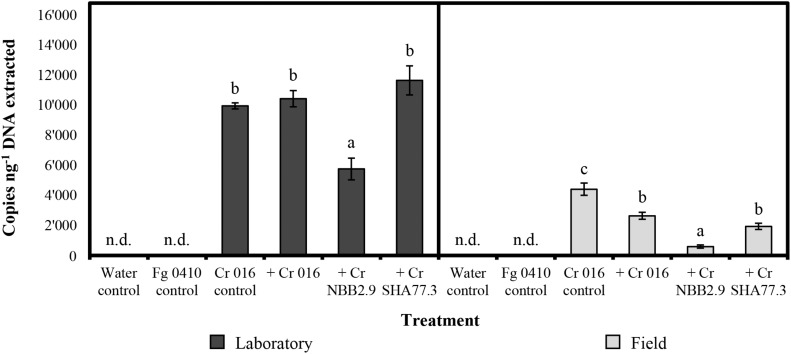
Quantification of *Clonostachys rosea* (Cr) by TaqMan qPCR in treated maize stalk samples after 10 weeks under laboratory or field conditions. Copies per ng of total DNA extracted determined in two repeated measurements. Bars show the mean copy number with vertical error bars for the standard error of the mean (*n* = 4). *Fusarium graminearum* (Fg) isolate 0410 was used for artificial infection 48 h before treatment. n.d. = not detected. For laboratory or field samples, the response variable “copies per ng DNA” was analyzed by one-way-analysis of variance (ANOVA) with “treatment” as the predicting factor (significance level = 0.05). Treatments within “laboratory” or “field” sharing the same letter are not significantly different according to a *post hoc* test (α = 0.05).

For the maize stalks that were infected with the pathogenic *F. graminearum* isolate 0410, the levels of detection ranged from 223 to 14′752 copies per ng of total DNA extracted, depending on the *C. rosea* strain applied and the incubation conditions during the experiment (Figure 2). On average, the detection level after incubation in the laboratory was 9′448 copies and thereby around four times higher than in the field with 2′387 copies, reflecting the more favorable growing conditions at 18°C with high relative humidity and microbial competition limited to *F. graminearum*. The highest mean level of detection with 11′653 copies was found when *C. rosea* strain SHA77.3 was applied on infected stalks and incubated in the laboratory, which was significantly (*p* < 0.001) higher than the number of copies from the treatment with strain NBB2.9, but not different compared with other strains. For the field samples, the positive control samples from stalks treated with strain 016 without previous *F. graminearum* inoculation, showed the highest detection level with a mean of 4′404 copies per ng DNA, which was significantly higher (*p*-value range: 0.007–<0.001) than the copy number from all other treatments. Under both incubation conditions, the strain NBB2.9 was detected at significantly (*p*-value range: 0.04–0.001) lower levels with means of 583 and 5′753 copies under field and laboratory conditions, respectively (Figure 2), suggesting a reduced colonization level of the infected maize stalks in the laboratory and in the field.

In the malting samples, *C. rosea* strain ACM941 was detected in significantly increasing levels over time after the inoculation at a concentration of 10^6^ conidia per kg for both barley batches, showing high (TS17-1; 3.16 ± 0.18 pg ng^–1^) and low (TS17-19; 0.70 ± 0.01 pg ng^–1^) levels of natural contamination with *Fusarium* spp. DNA (Table 4). Between the different malting stages, and for the batches TS17-1 and TS1-19, the mean number of copies per ng DNA increased from 1 to 53 (steeping → germination) and from 53 to 118 (germination → malt) or from 1 to 90 (steeping → germination) and from 90 to 209 (germination → malt), respectively. A strong increase was detected after 120 h of germination at 16°C, reflecting the time of incubation at the elevated grain moisture content of 45 ± 1%. Between the two different barley batches, detection levels were on average 75% higher in samples of TS17-19 that showed lower natural contamination with *Fusarium* spp. DNA.

**TABLE 4:** Quantification of *Clonostachys rosea* strain ACM941 by TaqMan qPCR in barley grains showing high or low natural contamination with *Fusarium* spp. DNA throughout the malting process.

**Malting stage**	**Activation**	**Steeping**	**Germination**	**Kilning**
Temperature	25°C	16°C	16°C	16°C–81°C
Time	24 h	27 h	120 h	21 h
Moisture content	30%	45%	45%	4%

**Sample**	**Mean copies *C. rosea* per ng extracted DNA ± STD**			

TS17-1 *Fusarium* sp. DNA: 3.16 ± 0.18 pg ng^–1^	0.38 ± 0.06^a^	1.34 ± 0.26^b^	52.60 ± 15.51^c^	118.00 ± 49.37^d^
TS17-19 *Fusarium* sp. DNA: 0.70 ± 0.01 pg ng^–1^	1.03 ± 0.24^a^	1.30 ± 0.13^a^	89.57 ± 40.01^a^	209.00 ± 26.66^b^

*Copies per ng of extracted DNA determined in two repeated measurements. For each batch, the response variable “copies per ng DNA” was analyzed by a Kruskal–Wallis test with “malting stage” as the predicting factor (significance level = 0.05). Mean copy numbers (*n* = 3) sharing the same letter are not significantly different according to a *post hoc* test (α = 0.05).*

## Discussion

The development of a robust method for the rapid and sensitive detection of the fungal antagonist *C. rosea* is highly useful to further explore its potential in pre- and post-harvest biological control of the pathogen and mycotoxin producer *F. graminearum*. The use of TaqMan qPCR, combining species-specific markers and fluorogenic probes is an established method to quantify and monitor mycotoxigenic fungi as pathogens (Waalwijk et al., 2004; Yli-Mattila et al., 2008) or at later stages as contaminants in foodstuff (Sarlin et al., 2006). In fact, TaqMan qPCR is also used for the detection and quantification of potential BCAs such as *T. harzianum* or *Paecilomyces lilacinus*, which helped to better understand their ability to colonize and suppress pathogens in the field (Atkins et al., 2005; López-Mondéjar et al., 2010). To our knowledge, only strain-specific markers are currently available for *C. rosea*, which were previously developed for the commercialized BCA *C. rosea* f. *catenulata* strain J1446 (Paavanen-Huhtala et al., 2000) and for *C. rosea* f. *rosea* strain GR5 (Bulat et al., 2000). The authors used universally primed-PCR (UP-PCR) and randomly amplified polymorphic DNA (RAPD) techniques to identify sequence-characterized amplified region (SCAR) markers. Recently, Legrand et al. (2018) applied these SCAR markers for J1446 to develop a qPCR assay and monitored the establishment of the BCA in non-sterile and sterilized soils artificially inoculated with *F. graminearum*. The interaction of the antagonist and the pathogen (also monitored by qPCR, using the RAPD markers of Nicholson et al., 1998) in the two environments, revealed significant growth inhibition of the pathogen by up to 50% when the co-introduced BCA was growing in sterilized soil, demonstrating the usefulness of sensitive quantification methods applicable to different matrices.

In the current study, we developed a TaqMan qPCR assay targeting the actin gene region to quantify *C. rosea*, including both intraspecific forms, *C. r.* f. *rosea* and *C. r.* f. *catenulata*, while maintaining a high specificity and sensitivity. The actin gene was chosen because of its stable expression in different tissues and cell types of *C. rosea* under a wide range of experimental conditions (Tzelepis et al., 2015; Zhang et al., 2017). Both, Demissie et al. (2018) and Nygren et al. (2018) used actin as the reference gene in expression studies and thereby demonstrated specific molecular responses by *C. rosea* in the interaction with *F. graminearum*. By using a synthetic DNA fragment comprising the amplified region and additional 50 bp to both 5′ and 3′ direction to prepare the standard curve, we determined a linear dynamic range between 10 and 10^6^ target copies with a limit of detection (LOD) of 100 copies per reaction. The efficiency of the amplification was consistently between 90 and 100% with *r*^2^ values > 0.99. Considering the single copy nature of the targeted gene region in the genome of *C. rosea* and the observation by Seh and Kenerley (1988) that its cells are predominantly uninucleate, the presented method provides a sensitive tool to monitor the presence and amount of the BCA in terms of genome copy numbers. While a multi copy target could further decrease the LOD of the PCR, the use of a single copy target may more accurately reflect the quantity of *C. rosea* cells within the sample. In addition, multi copy genes may suffer from significant variations in abundancy between isolates of the same species (Bilodeau et al., 2011). Hence, future investigations should aim to determine the relation between the number of genome copies and the presence of conidia or hyphae in treated substrates to enhance the extrapolation of the results towards actual fungal biomass.

The specificity of our assay was confirmed *in silico*, and subsequently against a diverse panel of fungi and bacteria by qPCR, including five *F. graminearum* strains and 12 different *Fusarium* species, other antagonists and against several other pathogens or saprophytes commonly associated with barley, maize or wheat. The fact that cross-reaction with the tested isolates of the closely related species *C. rhizophaga*, *C. pseudochloroleuca*, and *C. byssicola* was observed, but not with *C. rogersoniana*, is not surprising as the genus of *Clonostachys* is still not fully understood and several reclassifications of isolates or descriptions of new species have occurred over the past years (Abreu et al., 2014; Moreira et al., 2016). Our findings suggest that the tested isolates of *C. rosea*, *C. pseudochloroleuca*, and *C. rhizophaga* are highly conspecific within the targeted gene region. This is in support of Schroers (2001), who concludes that little morphological differences exist between the widely distributed *C. rosea* and the far more rarely isolated *C. rhizophaga*, and that their distinction relies mainly on phylogenetic analysis. The tropical species, *C. byssicola* and *C. pseudochloroleuca*, are more distinct due to the natural occurrence on decaying trees and a reportedly lower global distribution. Certainly, it is possible that environmental and especially soil samples may show a combined natural background of *C. rosea* and other *Clonostachys* species, which could interfere with absolute quantification. However, in the context of biological control of *F. graminearum* with repeated applications of conidia suspensions in large volumes, with more than 10^6^ colony forming units per ml, as proposed by Xue et al. (2014), a potential natural background of *Clonostachys* species on crop residues or in grains is likely to be negligible.

In order to validate the method with representative material, 96 different samples from maize crop residues and malted barley grain were extracted for DNA and then analyzed by qPCR. For the DNA isolation, we used a widely available DNA extraction kit that consists of a mechanical cell-lysis step and DNA purification by a spin column, developed to remove potential PCR inhibitors. Sarlin et al. (2006) previously evaluated this extraction method for high quality DNA, with barley samples in the development of a qPCR assay for *F. graminearum* quantification in malting. In the current study, we obtained reproducible results in terms of DNA quantity and quality. Furthermore, no PCR inhibitory effects were determined when adding increasing amounts of DNA isolated from maize stalks, barley or wheat grains up to 100 ng per PCR and then amplifying a spiked control. Such investigations of inhibition by co-extracted molecules is of crucial importance, as shifts in amplification due to partial or total PCR inhibition, result in striking changes of the calculated number of target copies or even false negative detections (Schena et al., 2013). We based our evaluation on a generally applicable protocol by Schneider et al. (2009) that was described to determine inhibitory effects on PCR. The authors showed that some soil matrices drastically inhibit qPCR at concentrations as low as 1 ng per reaction while others had no significant effect, adding up to 50 ng per reaction. Hence, it cannot be excluded that other sample matrices, which were not part of the present study, may have inhibitory effects. Still, the combination of using amplicon sequence-specific TaqMan qPCR and a short DNA fragment of only 156 bp favors a very high level of PCR efficiency, specificity and sensitivity (Schena et al., 2013). Alternatively, in cases where preliminary inhibition testing is not an option, future applications can rely on the preparation of matrix-matched standard curves that include dilutions of the DNA target and extracts of non-contaminated DNA from the respective plant or soil matrix.

This study revealed a qPCR approach for quantitative detection of different *C. rosea* strains, tested as antagonists against *F. graminearum* on crop residues. Previously, Palazzini et al. (2013) applied the *Fusarium*-specific TaqMan qPCR methods developed by Waalwijk et al. (2004) to monitor the population dynamics of *F. graminearum*, *F. avenaceum* and *F. verticillioides* in wheat crop residues treated with two different strains of *C. rosea*. The authors observed significant effects on the reduction of the pathogen DNA but were not drawing conclusions on differences in the presence of *C. rosea*, since at the time of the study, no qPCR method was available to correlate between the growth dynamics of the pathogens and the applied *C. rosea* strains. With the newly developed assay, a comparison between pathogen and antagonist DNA by qPCR from the same extract is feasible, even in a combined form of a multiplex analysis.

The analysis of the malting barley DNA samples gave first preliminary evidence on the growing potential of *C. rosea* within a small scale malting process. This is comparable to the growth potential of several *Fusarium* species, including *F. graminearum*, as previously studied in the same process by Virkajärvi et al. (2017). The authors determined the amount of *Fusarium* sp. DNA present before and after the malting process to characterize the fungal dynamics, but also correlated the presence of different *Fusarium* species with the production of gushing inducing hydrophobins. These small surface-active proteins can be extensively produced by several *Fusarium* species (Sarlin et al., 2012) and interact with CO_2_-molecules causing the spontaneous and undesirable gushing of carbonated beverages. It is well known that *F. graminearum* and other mycotoxin producers can grow especially between the steeping and germination of the grain, when high relative humidity (∼45%) and temperatures between 14 and 18°C provide favorable conditions for microbial growth (Schwarz, 2017). As a preventive measure, Laitila et al. (2007) showed that the introduction of an antagonistic yeast together with lactic acid bacteria, both able to proliferate and counteract the pathogen during malting, significantly improved the final quality of the malt. In their study, both microbes were clearly associated with the industrial process of barley malting. This newly developed assay can be applied to monitor also the presence of an exogenous organism that may be present in the malting material prior to processing, or after *C. rosea* was deliberately introduced into the malting process to suppress *F. graminearum*.

## Conclusion

For the detection and quantification of *C. rosea*, we present a practical and sensitive alternative to culture-dependent methods. Within the scope of improving the biological control of the noxious fungal pathogen *F. graminearum*, we suggest its application to monitor the growth dynamics of the BCA *C. rosea*, when applied in sustainable disease control strategies.

## Data Availability

All datasets generated for this study are included in the manuscript and/or the Supplementary Files.

## Author Contributions

The presented work was a collaborative effort between the VTT Technical Research Centre of Finland (VTT) and the Research Division Plant Protection of Agroscope (Agroscope). AG, ES, TP, JL, and AL planned and conceived the experiments in Finland. SV and AL supervised the study. AG led the writing of the manuscript with ES, TP, SV, and AL contributing in the preparation of the manuscript. BK provided valuable feedback on the experimental work and the final version of the manuscript.

## Conflict of Interest Statement

AL, ES, JL, and TP were employed by the VTT Technical Research Centre of Finland Ltd.

The remaining authors declare that the research was conducted in the absence of any commercial or financial relationships that could be construed as a potential conflict of interest.

## Footnotes

1 https://genome.jgi.doe.gov/cgi-bin/dispGeneModel?db=Cloro1&id=114937

2 https://blast.ncbi.nlm.nih.gov/Blast.cgi

3 https://www.ebi.ac.uk/Tools/msa/clustalo/

4 https://www.ncbi.nlm.nih.gov/tools/primer-blast/index.cgi?LINK_LOC=BlastHome

5 https://www.geneious.com/
