# Corrigendum: TaqMan qPCR for Quantification of *Clonostachys rosea* Used as a Biological Control Agent Against *Fusarium graminearum*

**DOI:** 10.3389/fmicb.2019.01839

**Published:** 2019-08-13

**Authors:** Alejandro Gimeno, Elina Sohlberg, Tiina Pakula, Jenni Limnell, Beat Keller, Arja Laitila, Susanne Vogelgsang

**Affiliations:** ^1^Ecological Plant Protection in Arable Crops, Research Division Plant Protection, Agroscope, Zurich, Switzerland; ^2^Molecular Plant Biology and Phytopathology, Department of Plant and Microbial Biology, University of Zurich, Zurich, Switzerland; ^3^VTT Technical Research Centre of Finland Ltd., Espoo, Finland

**Keywords:** qPCR, *Clonostachys rosea*, biological control agent, *Fusarium graminearum*, Fusarium head blight, MycoKey

In the original article, there was a mistake in Table 2 as published. The primer sequence (5′-3′) of *VTTact*—reverse contained a false nucleobase. The corrected Table 2 appears below.

**Table 2:** Primer and hydrolysis probe sequences (5′–3′) for the specific amplification of *Clonostachys rosea* with TaqMan qPCR.

**Primer/probe**	**Sequence (5^**′**^to 3^**′**^)**
*VTTact*-forward	GGCCAGAGATTGTGTTGATGA
*VTTact*-reverse	ACAGGTTAGGCTCAATGCTC
*VTTact* probe	GAGGCTGGCAAGAGAGGTCAGTCAC

The authors apologize for this error and state that this does not change the scientific conclusions of the article in any way. The original article has been updated.

