# Supplementary material for: TaqMan qPCR for Quantification of Clonostachys rosea Used as a Biological Control Agent Against Fusarium graminearum
Source: Front Microbiol. 2019 Jul 16;10:1627. doi: 10.3389/fmicb.2019.01627 (PMC6646457; doi:10.3389/fmicb.2019.01627)
Supplement: Supplementary file 1 [file Data_Sheet_1.pdf]

Table S1 | Amplification specificity of the TaqMan qPCR – Part 1. For each DNA extract, two individual reactions were performed using 10 ng of total DNA (5 µl).
Clonostachys rosea f. *rosea*: 016; VTT D-97674; CCOS 1864; CCOS 1865; VTT D-161647; VTT D-96593. *Clonostachys rosea* f. *catenulata*: VTT D-97673; VTT D-95548. *Clonostachys pseudochloroleuca*: CBS 187.94 T. *Clonostachys rhizophaga*: CCOS 1863; CBS 125416. *Clonostachys rogersoniana*: CBS 920.97 T.
">, E - Late Ct call" = cutoff value for the method.

Experiment: Specificity *C. rosea* Selected Filter: FAM (465-510)						
Include	Color	Pos	Name	Ct	Concentration	Standard
TRUE	128	A1	Std1	17.72	3.07E+06	3700000
TRUE	128	B1	Std1	17.44	3.67E+06	3700000
TRUE	128	C1	Std1	16.15	8.34E+06	3700000
TRUE	128	D1	Std2	21.28	3.21E+05	370000
TRUE	128	E1	Std2	21.08	3.63E+05	370000
TRUE	128	F1	Std2	20.45	5.42E+05	370000
TRUE	128	A2	Std3	23.80	6.47E+04	37000
TRUE	128	G1	Std3	24.50	4.13E+04	37000
TRUE	128	H1	Std3	24.88	3.24E+04	37000
TRUE	128	B2	Std4	28.82	2.66E+03	3700
TRUE	128	C2	Std4	28.24	3.84E+03	3700
TRUE	128	D2	Std4	28.51	3.25E+03	3700
TRUE	128	E2	Std5	31.71	4.26E+02	370
TRUE	128	F2	Std5	31.85	3.88E+02	370
TRUE	128	G2	Std5	32.02	3.49E+02	370
TRUE	255	A3	016	19.93	7.52E+05	
TRUE	255	A4	016	20.09	6.80E+05	
TRUE	255	A5	VTT D-95548	20.79	4.36E+05	
TRUE	255	A6	VTT D-95548	20.74	4.50E+05	
TRUE	65280	A7	VTT D-82182			
TRUE	65280	A8	VTT D-82182			
TRUE	65280	A9	VTT E-93497			
TRUE	65280	A10	VTT E-93497			
TRUE	65280	A11	1145			
TRUE	65280	A12	1145			
TRUE	255	B3	VTT D-161647	20.22	6.26E+05	
TRUE	255	B4	VTT D-161647	20.33	5.83E+05	
TRUE	65280	B5	VTT D-95470			
TRUE	65280	B6	VTT D-95470			
TRUE	65280	B7	VTT D-80141			
TRUE	65280	B8	VTT D-80141			
TRUE	65280	B9	VTT D-131555			
TRUE	65280	B10	VTT D-131555			
TRUE	65280	B11	VTT D-071272			
TRUE	65280	B12	VTT D-071272			
TRUE	255	C3	CCOS 1865	20.08	6.84E+05	
TRUE	255	C4	CCOS 1865	20.10	6.76E+05	
TRUE	255	C5	CBS 125416	21.20	3.36E+05	
TRUE	255	C6	CBS 125416	21.22	3.32E+05	
TRUE	65280	C7	VTT D-82087			
TRUE	65280	C8	VTT D-82087			
TRUE	65280	C9	VTT D-94433			
TRUE	65280	C10	VTT D-94433			
TRUE	65280	C11	VTT D-161648			
TRUE	255	C12	VTT D-161648	>35.00	>, E - Late Ct call	

TRUE	255	D3	CCOS 1864	20.37	5.70E+05
TRUE	255	D4	CCOS 1864	20.44	5.45E+05
TRUE	255	D5	CBS 187.94 T	21.37	3.01E+05
TRUE	255	D6	CBS 187.94 T	21.14	3.49E+05
TRUE	65280	D7	VTT D-77057		
TRUE	65280	D8	VTT D-77057		
TRUE	65280	D9	VTT D-80148		
TRUE	65280	D10	VTT D-80148		
TRUE	65280	D11	VTT D-131559		
TRUE	65280	D12	VTT D-131559		
TRUE	255	E3	CCOS 1863	20.66	4.75E+05
TRUE	255	E4	CCOS 1863	20.89	4.09E+05
TRUE	65280	E5	CBS 920.97 T		
TRUE	65280	E6	CBS 920.97 T		
TRUE	65280	E7	VTT D-96653		
TRUE	65280	E8	VTT D-96653		
TRUE	65280	E9	VTT D-72014		
TRUE	65280	E10	VTT D-72014		
TRUE	65280	E11	VTT D-80134		
TRUE	65280	E12	VTT D-80134		
TRUE	255	F3	VTT D-97674	21.48	2.81E+05
TRUE	255	F4	VTT D-97674	21.51	2.76E+05
TRUE	65280	F5	CBS 121292		
TRUE	65280	F6	CBS 121292		
TRUE	65280	F7	VTT D-76039		
TRUE	65280	F8	VTT D-76039		
TRUE	65280	F9	VTT D-03931		
TRUE	65280	F10	VTT D-03931		
TRUE	65280	F11	VTT D-99750		
TRUE	65280	F12	VTT D-99750		
TRUE	255	G3	VTT D-96593	21.51	2.76E+05
TRUE	255	G4	VTT D-96593	22.02	2.00E+05
TRUE	65280	G5	2113		
TRUE	65280	G6	2113		
TRUE	65280	G7	VTT D-94425		
TRUE	65280	G8	VTT D-94425		
TRUE	65280	G9	11132		
TRUE	65280	G10	11132		
TRUE	65280	G11	VTT D-76046		
TRUE	65280	G12	VTT D-76046		
TRUE	65280	H2	-	-	-
TRUE	255	H3	VTT D-97673	21.17	3.43E+05
TRUE	255	H4	VTT D-97673	21.12	3.53E+05
TRUE	65280	H5	335		
TRUE	65280	H6	335		
TRUE	65280	H7	VTT E-78076		
TRUE	65280	H8	VTT E-78076		
TRUE	65280	H9	VTT D-76038		
TRUE	65280	H10	VTT D-76038		
TRUE	65280	H11	Water		
TRUE	255	H12	Water	>35.00	>, E - Late Ct call

Table S1 | Amplification specificity of the TaqMan qPCR – Part 2. For each DNA extract, two individual reactions were performed using 10 ng of total DNA (5 µl).
Clonostachys byssicola: CBS 364.78. “>, E - Late Ct call” = cutoff value for the method.

Experiment: Specificity *C. rosea* Selected Filter: FAM (465-510)						
Include	Color	Pos	Name	Ct	Concentration	Standard
TRUE	128	A1	Std1	17.15	2.51E+06	3700000
TRUE	128	B1	Std1	16.33	3.93E+06	3700000
TRUE	128	C1	Std1	15.11	7.71E+06	3700000
TRUE	128	D1	Std2	20.01	5.19E+05	370000
TRUE	128	E1	Std2	20.87	3.24E+05	370000
TRUE	128	F1	Std2	21.74	2.01E+05	370000
TRUE	128	A2	Std3	24.69	3.98E+04	37000
TRUE	128	G1	Std3	24.50	4.40E+04	37000
TRUE	128	H1	Std3	24.84	3.66E+04	37000
TRUE	128	B2	Std4	24.57	4.24E+04	3700
TRUE	128	C2	Std4	28.32	5.38E+03	3700
TRUE	128	D2	Std4	28.55	4.75E+03	3700
TRUE	128	E2	Std5	31.58	8.97E+02	370
TRUE	128	F2	Std5	31.98	7.21E+02	370
TRUE	128	G2	Std5	32.04	6.96E+02	370
TRUE	65280	A3	VTT D-00808			
TRUE	65280	A4	VTT D-00808			
TRUE	255	A5	CBS 364.78	30.48	1.65E+03	
TRUE	255	E5	CBS 364.78	30.59	1.55E+03	
TRUE	255	B5	CBS 364.78 (1:10)	34.10	2.25E+02	
TRUE	255	F5	CBS 364.78 (1:10)	>35.00	>, E - Late Ct call	
TRUE	255	C5	CBS 364.78 (1:100)	>35.00	>, E - Late Ct call	
TRUE	255	G5	CBS 364.78 (1:100)	>35.00	>, E - Late Ct call	
TRUE	65280	C3	VTT D-76024			
TRUE	65280	C4	VTT D-76024			
TRUE	65280	B3	VTT D-94422			
TRUE	65280	B4	VTT D-94422			
TRUE	65280	D3	VTT D-79121			
TRUE	65280	D4	VTT D-79121			
TRUE	65280	E3	*Hordeum vulgare* L.			
TRUE	65280	E4	*Hordeum vulgare* L.			
TRUE	65280	F3	VTT D-051089T			
TRUE	255	F4	VTT D-051089T	>35.00	>, E - Late Ct call	
TRUE	65280	G3	VTT D-82082			
TRUE	65280	G4	VTT D-82082			
TRUE	65280	H2	-	-	-	
TRUE	65280	H3	VTT D-76042			
TRUE	65280	H4	VTT D-76042			
TRUE	65280	D5	Water			
TRUE	65280	H5	Water			
TRUE	65280	B4	VTT E-90398			
TRUE	65280	B5	VTT E-90398			
TRUE	255	C10	VTT C-92011	>35.00	>, E - Late Ct call	
TRUE	65280	C11	VTT C-92011			
TRUE	65280	H8	VTT D-03923			
TRUE	65280	H9	VTT D-03923			
