# Supplementary figures and images for: TaqMan qPCR for Quantification of Clonostachys rosea Used as a Biological Control Agent Against Fusarium graminearum
Source: Front Microbiol. 2019 Jul 16;10:1627. doi: 10.3389/fmicb.2019.01627 (PMC6646457; doi:10.3389/fmicb.2019.01627)

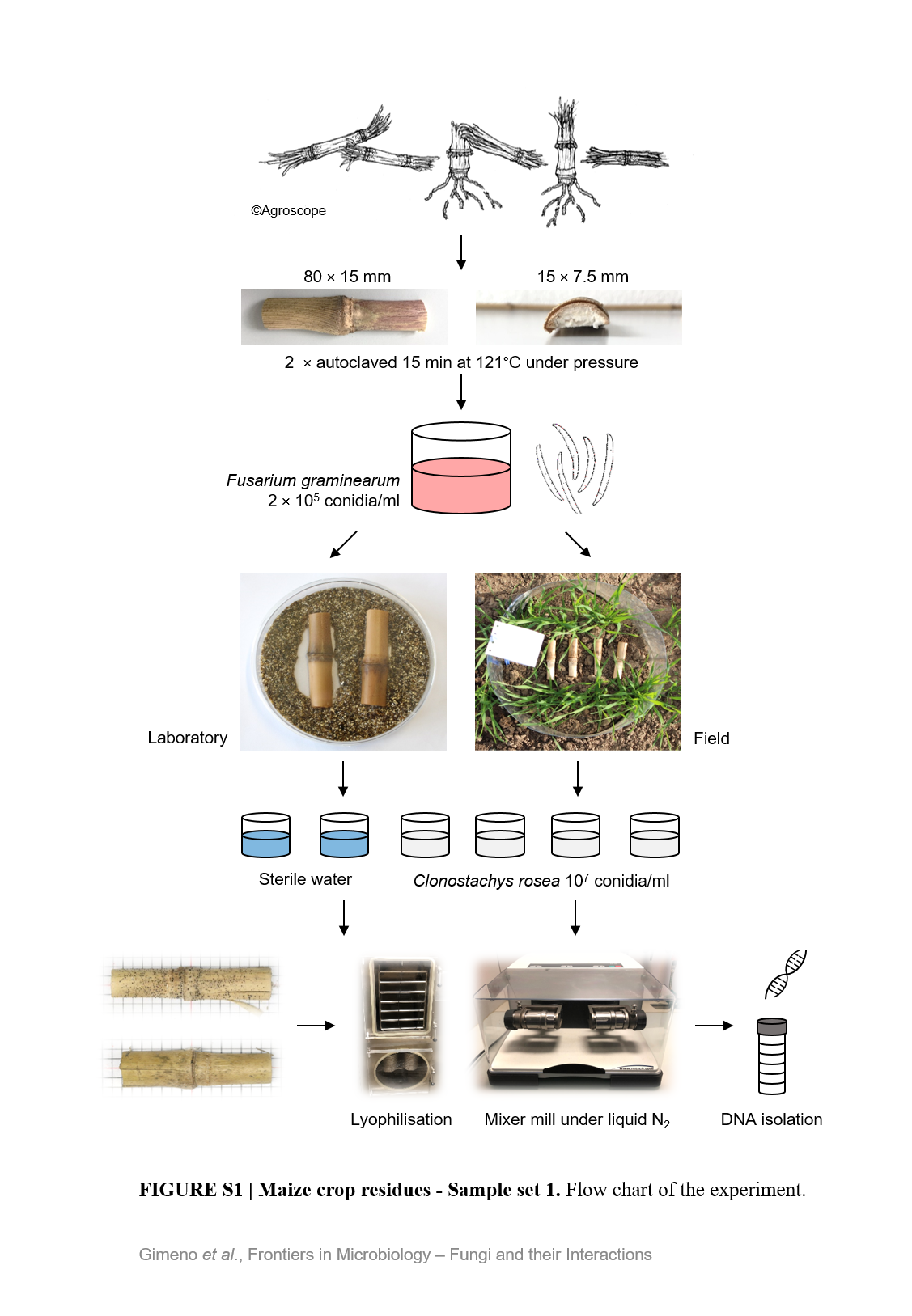

Supplement: Supplementary file 2 [file Image_1.TIF]

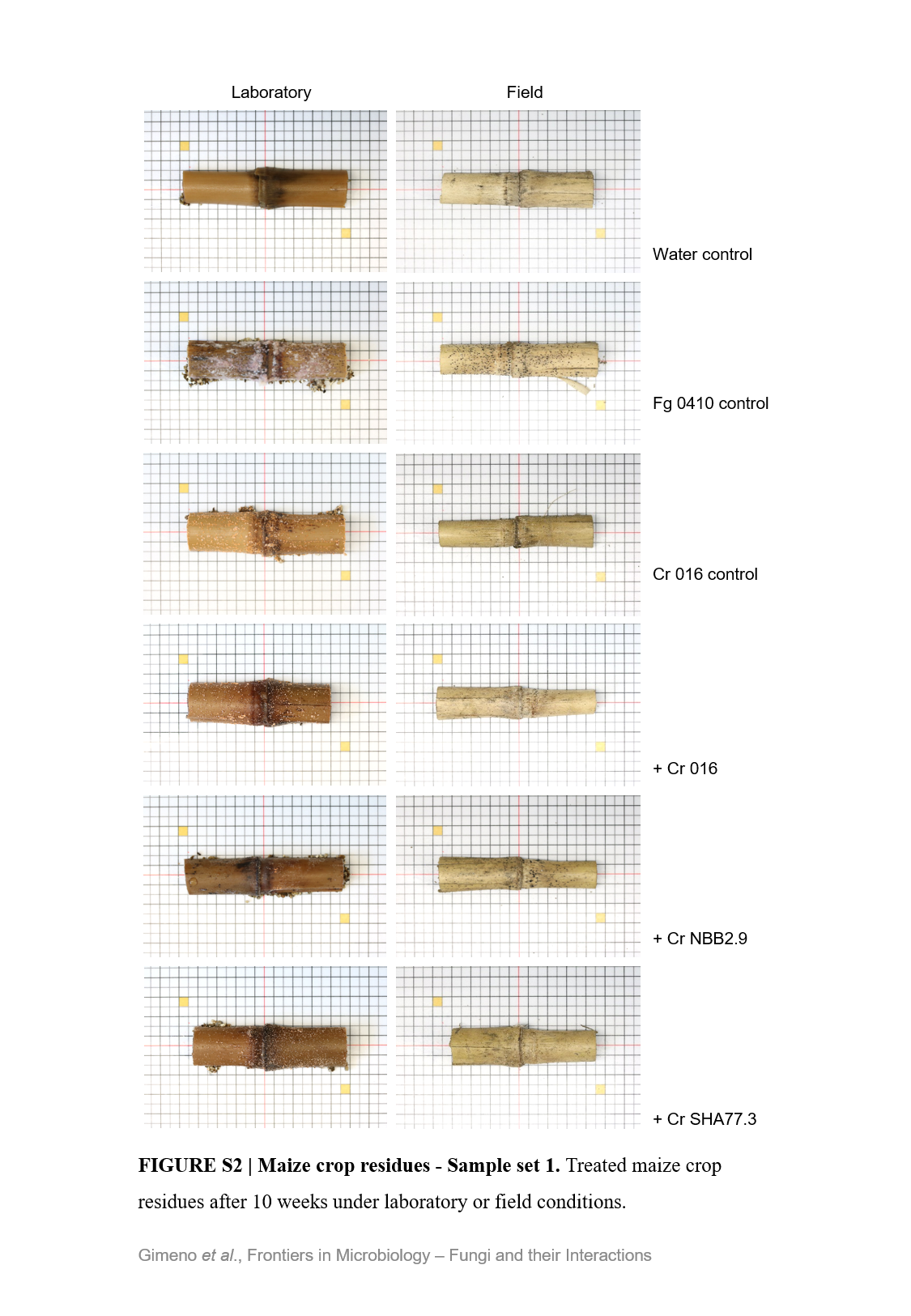

Supplement: Supplementary file 3 [file Image_2.TIF]

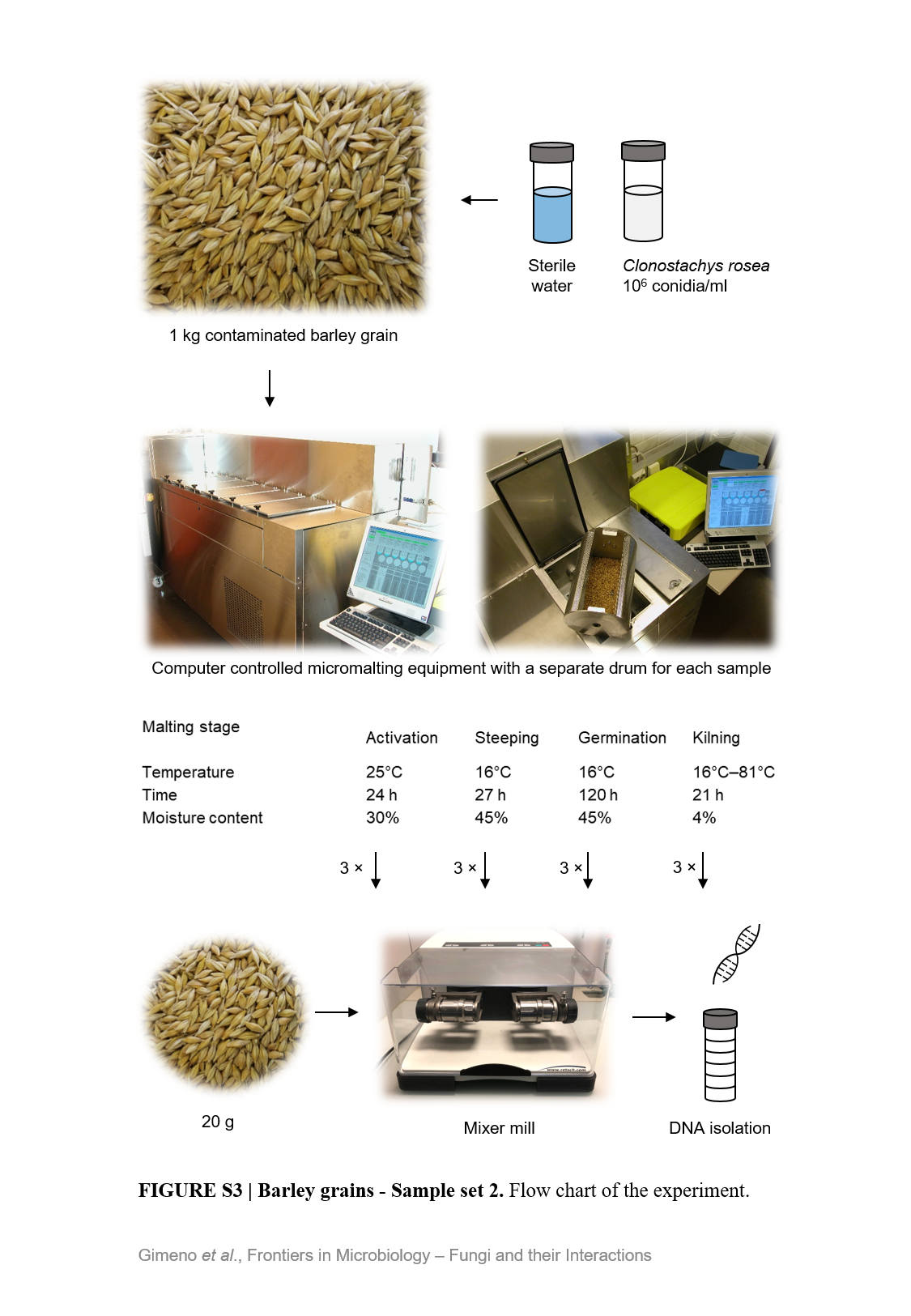

Supplement: Supplementary file 4 [file Image_3.TIF]

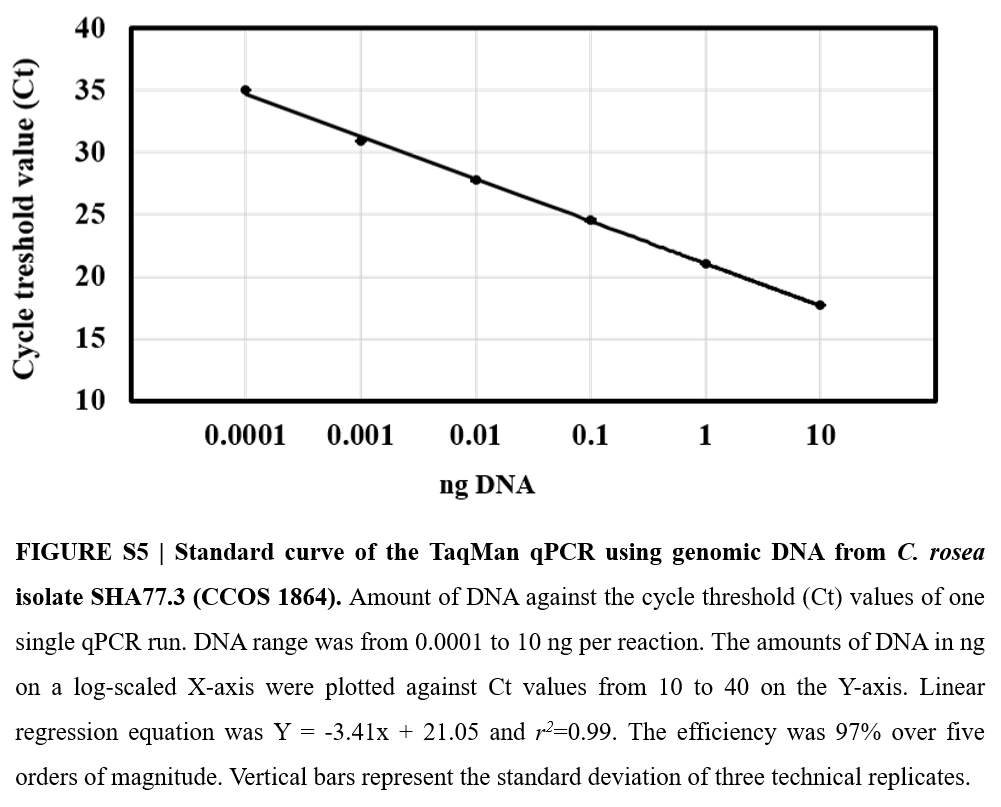

Supplement: Supplementary file 6 [file Image_5.tif]
