# Supplementary material for: TaqMan qPCR for Quantification of Clonostachys rosea Used as a Biological Control Agent Against Fusarium graminearum
Source: Front Microbiol. 2019 Jul 16;10:1627. doi: 10.3389/fmicb.2019.01627 (PMC6646457; doi:10.3389/fmicb.2019.01627)
Supplement: Supplementary file 5 [file Image_4.pdf]

A

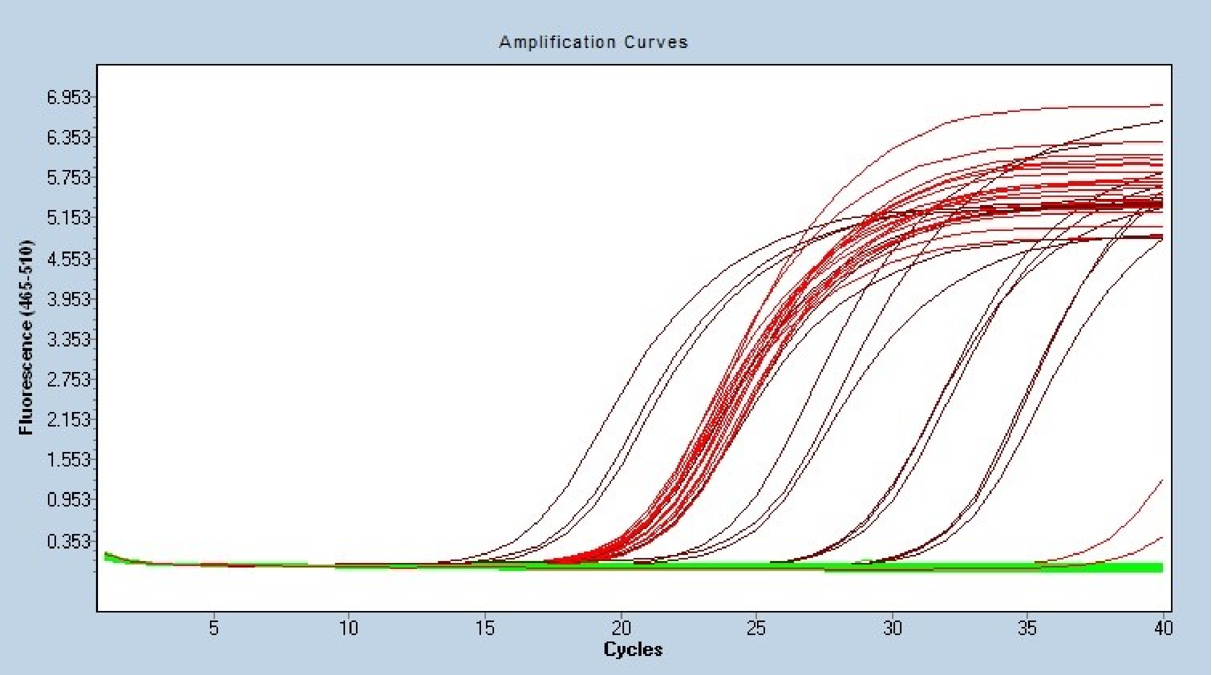

B

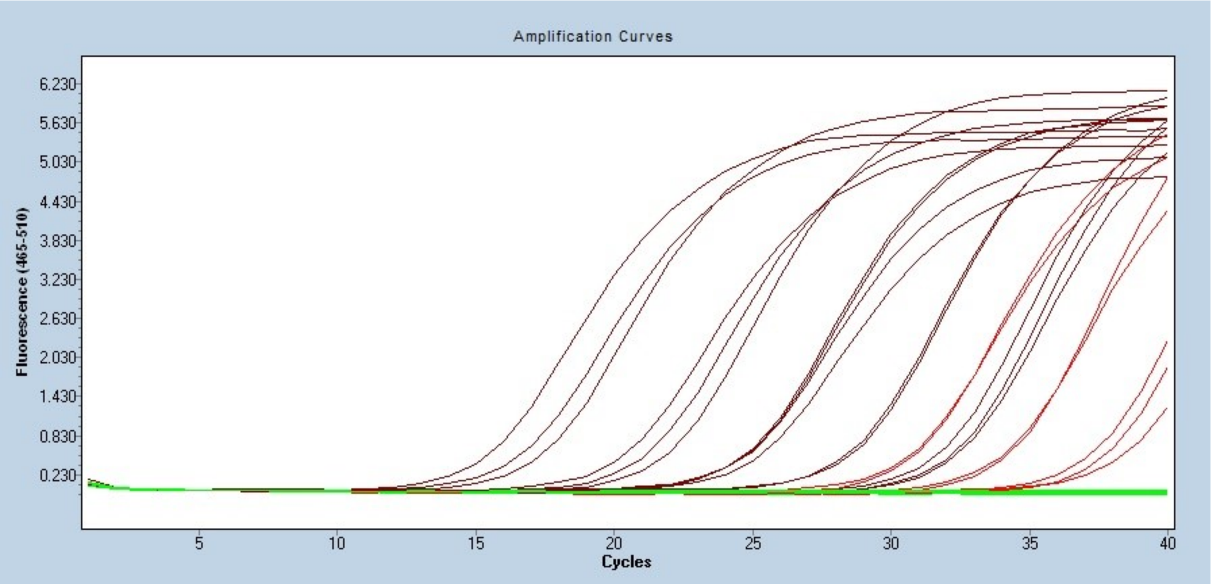

Figure S4 | Amplification curves of the TaqMan qPCR. For each DNA extract, two individual reactions were performed using 10 ng of total DNA (5 µl) and the amplification was examined. (A) Part 1 and (B) Part 2. >35.00 = cutoff value for the method.
